# Healthy younger and older adults control foot placement to avoid small obstacles during gait primarily by modulating step width

**DOI:** 10.1186/1743-0003-9-69

**Published:** 2012-10-03

**Authors:** Brian W Schulz

**Affiliations:** 1 VA HSR&D/RR&D Center of Excellence, Maximizing Rehabilitation Outcomes, James A. Haley VA Hospital, Tampa, FL, USA

**Keywords:** Foot placement, Gait variability, Gait control, Adaptive gait, Obstacles, Strategy selection, Locomotion, Ambulation, Biomechanics

## Abstract

**Background:**

Falls are a significant problem in the older population. Most falls occur during gait, which is primarily regulated by foot placement. Variability of foot placement has been associated with falls, but these associations are inconsistent and generally for smooth, level flooring. This study investigates the control of foot placement and the associated gait variability in younger and older men and women (N=7/group, total N=28) while walking at three different speeds (slow, preferred, and fast) across a control surface with no obstacles and surfaces with multiple (64) small (10cm long ×13mm high) visible and hidden obstacles.

**Results:**

Minimum obstacle distance between the shoe and nearest obstacle during each footfall was greater on the visible obstacles surface for older subjects because some of them chose to actively avoid obstacles. This obstacle avoidance strategy was implemented primarily by modulating step width and to a lesser extent step length as indicated by linear regressions of step width and length variability on minimum obstacle distance. Mean gait speed, step length, step width, and step time did not significantly differ by subject group, flooring surface, or obstacle avoidance strategy.

**Conclusions:**

Some healthy older subjects choose to actively avoid small obstacles that do not substantially perturb their gait by modulating step width and, to a lesser extent, step length. It is not clear if this obstacle avoidance strategy is appropriate and beneficial or overcautious and maladaptive, as it results in fewer obstacles encountered at a consequence of a less efficient gait pattern that has been shown to indicate increased fall risk. Further research is needed on the appropriateness of strategy selection when the environmental demands and/or task requirements have multiple possible completion strategies with conflicting objectives (i.e. perceived safety vs. efficiency).

## Background

Falls are a significant problem in the older population and are responsible for nearly half of all injury-related deaths in persons over the age of 65 [1] and most of these falls (67%) occur during gait [2]. Modulation of foot placement is the primary mechanism by which humans regulate and restore dynamic balance during gait [3]. Increased spatial and temporal variability of foot placement on smooth, level surfaces has been associated with older age [4,5] and increased fall risk [6-8], but the direction of the effects and which measure of variability is most predictive remains unclear. For example, Hausdorff et al. found that increased step time variability prospectively discriminated fallers from non-fallers [7], while Owings and Grabiner found step width variability to be a more meaningful descriptor of locomotion control than step time variability [4]. Increased variability in step width has been associated with older age [4,5], but Maki found that decreased step width variability combined with increased step width had a greater predictive power to discriminate fallers from non-fallers than step time variability [8]. Even more ambiguously, Brach et al. [9] found no association of step time variability with recent fall history and that either too much or too little step width variability was associated with recent fall history, but only in subjects who walked at or near normal gait speeds. The finding that step variability decreases with increasing gait speed further confound these effects [10], as older and more impaired populations often walk slower, which in itself increases gait variability.

Population-specific impairments may result in increased gait variability across flat, smooth, level surfaces, but environmental demands may also require increases in gait variability to avoid obstacles or other threats. Stepping over a single discrete obstacle has been well-characterized [11] and gait across uneven or challenging surfaces with multiple obstacles has also been investigated [12-14]. However, these studies did not control for gait speed, which is known to affect many gait parameters [10,15,16], and partially or completely hid the obstacles on the experimental surfaces used and thus could not investigate visually-controlled feedforward adaptations. Furthermore, these studies did not track the location of the feet in relation to all of the obstacles on the surface to quantify obstacle avoidance strategies. This study investigated the control of foot placement by unimpaired younger and older adults when walking across surfaces with visible and hidden obstacles at three different speeds.

As per the earlier findings of Thies et al. in healthy younger and older women [12], I hypothesized that the variability of step width and time would be greater on uneven surfaces and that step width variability would be greater in the older subjects. I also hypothesized that subjects would actively avoid obstacles on the surface with visible obstacles (as indicated by greater minimum distances from the shoe sole to the nearest obstacle) as compared to the surfaces with no obstacles and hidden obstacles and that this avoidance would be accomplished by greater gait variability (standard deviations of step length, width, and time) on the surface with visible obstacles as compared to the surfaces with no obstacles and hidden obstacles.

## Methods

### Subjects and instrumentation

A convenience sample of younger (defined by age≤35 years; range was 20–35; mean±standard deviation (SD) was 27±5 years) and older (defined by age≥65 years; range was 66–82; mean±SD was 72±5 years) unimpaired men and women were recruited from the community. Subjects were evenly split by gender within age group to test for gender effects in a parallel study reported elsewhere. While no gender effects were hypothesized, data were tested by subject group (including gender) to better display effects. Seven subjects were in each of the four age-gender groups, for a total *n* of 28. These subjects had no major health problems, 20/20 corrected vision, and good balance as defined by one-legged stance times of over 30 seconds on each foot. Human subject research oversight for this project was provided by the University of South Florida Institutional Review Board (study number 106090) in compliance with the Helsinki Declaration. After completing the informed consent process to authorize data collection and publication, each subject was given instrumented shoes in their own size (model 811, New Balance Athletic Shoe, Inc., Boston, MA, USA).

The shoes and floor were instrumented and digitized as described in detail previously [17] and briefly summarized here. Each experimental shoe had eight markers mounted on short threaded rods partially embedded in the outsole, with four affixed to the toe and four to the heel. The markers and sole of each shoe, the flooring surfaces, and obstacles were digitized prior to subject testing. A 13-camera Vicon MX40 system using Workstation v5.2.9 was used to collect all data at 120Hz (Vicon, Centennial, CO, USA).

### Data collection

Data were collected as previously reported [17], but these methods are briefly described here. Each subject performed four gait passes at three speeds (“slower than preferred”, “preferred”, and “as fast as safely possible”) across three 1.22m (4 foot) wide × 4.88m (16 foot) long surfaces. The three floor surfaces traversed were “no obstacles” (flat, smooth, level surface), “visible obstacles” (white obstacles on black surface), and “hidden obstacles” (black obstacles on black surface). Surface conditions were presented in randomized order and speed conditions were randomized within each surface condition. Subjects started and stopped walking from a position 1.5-1.9m outside of the capture volume so they were travelling at or close to a steady-state speed while walking on the flooring surfaces. A safety harness was worn at all times to minimize the chance of fall-related injuries.

All trials were conducted under identical low lighting conditions designed to minimize visual feedback of obstacle location for the hidden obstacles surface condition (complete details in [17]). Subjects were not allowed to see the hidden obstacles surface under normal lighting conditions and were instructed that the obstacles on this surface were identical to the visible obstacles in size, shape, and number, but that their layout was only “similar” while in fact their layout was identical.

No specific instructions regarding gaze were given for the no obstacles conditions. Subjects were instructed that they may look at the floor during the visible obstacles condition, but that they should keep their eyes focused on the end of the walkway for the hidden obstacles condition. These differing instructions for the two obstacle conditions were adopted as a countermeasure to subject attempts to see the hidden obstacles (by leaning forward and looking downward) and were intended to maintain consistent posture throughout all surface conditions. If a subject asked for clarification on how they were “supposed to walk across this floor”, they were instructed to walk across the floor as they would normally walk across a similar surface outside of a laboratory.

The obstacles were 10cm lengths cut from roughly triangular wood stock (locally sold as “cove molding”). The cross section of this stock is roughly a right triangle that resulted in ~13mm high obstacles when attached to the floor by the larger “hypotenuse” face with the 90° angle edge facing upwards. These obstacles were affixed such that the visible and hidden obstacles floor surfaces each had 64 obstacles affixed in the same random configuration (obstacle density of 10.76/m^2^ or 1/ft^2^). The experimental floor surfaces were attached to the floor of the laboratory in the same repeatable positions via a combination of locating pins, alignment rails, hold down pins, and high-strength neodymium magnets that pulled the plywood floor surface sections tightly against the steel-panel raised flooring system that comprised the laboratory floor.

### Data processing

All data were collected and labelled using Vicon Workstation, then processed using Visual3D (C-Motion, Inc., Germantown, MD, USA) and custom MATLAB code (The MathWorks, Natick, MA, USA). Motion capture data were low-pass filtered using a zero-lag 4th order Butterworth filter at 10Hz, as determined from prior work and spectral analysis of pilot data.

The initial processing of the shoe sole and floor data were as per previously-described methods for calculating minimum toe clearance [17]. However, the final processing differed in that these data were used to calculate the minimum distance between any digitized point on the shoe sole and any obstacle (whether present on the testing surface or not) during each footfall.

### Statistical analysis

The first of the nine dependent variables tested here was the minimum distance from either shoe to any obstacle (minimum obstacle distance) during each footfall. As the distribution of minimum obstacle distance for all steps for a given gait condition was not normally distributed, the median rather than the mean of all values during a single gait condition was used. Four spatiotemporal gait parameters were also examined to determine how foot placement was controlled: gait speed during each trial, step length, step width, and step time. The central tendency (mean) and variability (standard deviation) of each of these four gait parameters were tested. All spatial gait parameters were normalized to mean leg length (shank length + thigh length) and right and left foot values were combined for all applicable variables.

The distributions of minimum obstacle distance and all variability measures were skewed (skewness ≥ 2, Shapiro-Wilks p<0.0001), so the Kruskall-Wallis one-way analysis of variance by ranks was used to independently test for the effects of subject group (younger men, younger women, older men, or older women), floor surface (no obstacles, visible obstacles, or hidden obstacles), and instructed gait speed (slow, preferred, or fast), on these variables. Repeated-measures linear mixed models were used to simultaneously test the effects of subject group, floor surface, and instructed gait speed on the remaining five dependent variables. Uncorrected p-values were reported but all thresholds of significance were corrected as per Bonferroni – main effects considered significant at p<0.0031 (0.05/(9 dependent variables × 4 subject groups × 3 speeds × 3 surfaces)). If main effects for speed or surface were determined to be significant, post-hoc multiple comparisons were conducted and considered significant at p<0.017 (0.05/3 speeds or surfaces). SAS (SAS Institute Inc., Carey, NC, USA) was used for all statistical analyses.

## Results

### Minimum obstacle distance

**Figure 1:**
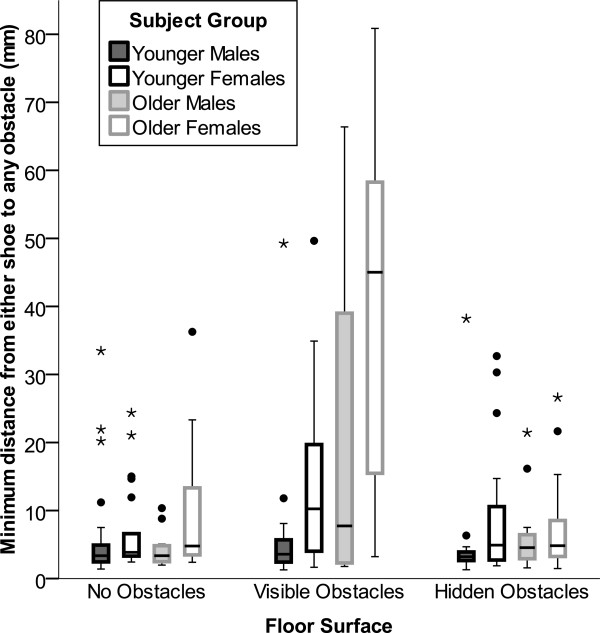
**Boxplot of minimum obstacle distance by subject group and floor surface.** Minimum distances between any point on the shoe and any of 64 small obstacles on the floor during each footfall was significantly greater for older subjects (p<0.0001) and on the visible obstacle floor (p<0.0001) primarily due to older women and some older men actively avoiding visible obstacles. Dots indicate values between 1.5 and 3*interquartile range and stars indicate values >3*interquartile range.

Testing was well tolerated by all subjects and only one older male subject stumbled due to contact with an obstacle (fast gait speed on visible obstacles surface). He recovered within one or two steps and these recovery steps were excluded from analysis. Data variability did not appear to be affected by the fewer steps recorded for faster speeds and taller subjects with longer legs. Significant minimum obstacle distance effects were found for subject group (p<0.0001) and floor surface (p<0.0001) that were mostly due to greater minimum obstacle distance by older subjects –particularly the older women – on the visible obstacles surface (Figure 1).

**Figure 2:**
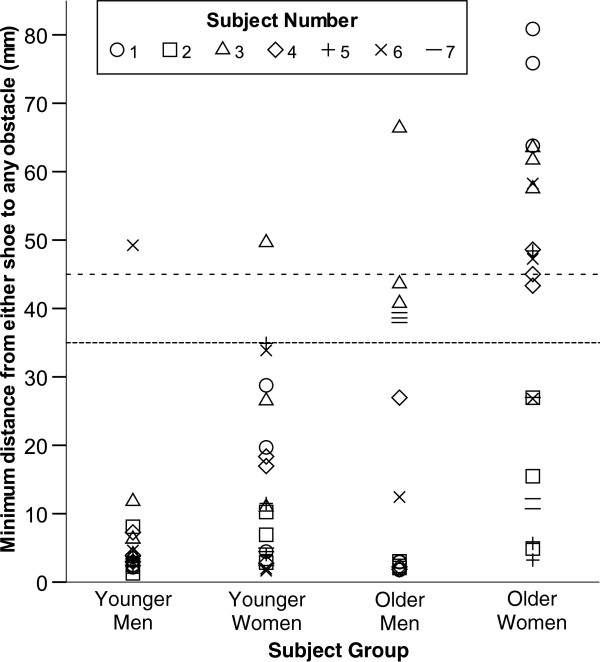
**Thresholded scatterplot of individual subject minimum obstacle distances on visible obstacles surface by subject group.** Median of the minimum obstacle distance for all steps at each instructed gait speed (slow, preferred, and fast) on the surface with visible obstacles. As gait speed is not indicated on this figure, three points are shown for each subject. If a 35mm threshold (tightly-spaced dashes) is applied to categorize subjects as “normal foot placement” or “active avoiders”, 29% of older men (all speeds for two subjects) and 57% of older women (all speeds for three subjects and one or two speeds for two others) would be considered to be actively avoiding the obstacles. If this threshold is raised to 45mm (loosely-spaced dashes) then 52% of the older females (one less speed for subject 4) would still be considered to be actively avoiding the obstacles while all but one of the older male conditions would not. Five percent of both younger subject groups and older men (preferred speed for one subject from each group) had a minimum obstacle distance value above both thresholds.

The greater minimum obstacle distance values of the older subjects on the visible obstacles surface were inconsistent across subjects and appeared to be the result of different strategy selection (i.e. a bimodal distribution) by specific subjects rather than a consistent increase in minimum obstacle distance within or between subject groups (Figure 2). If a threshold is applied to the minimum obstacle distance data to categorize subjects as “normal foot placement” or “actively avoiding obstacles”, only 5% of younger men and women (preferred speed for one subject in each group) had a minimum obstacle distance on the visible obstacles >35mm while 29% of older men (all speeds for two subjects) and 57% of older women (all speeds for three subjects and one or two speeds for two others) had a minimum obstacle distance >35mm (Figure 2). If this threshold is raised to 45mm, then 52% of the older women (one less speed for subject 4) are still actively avoiding the obstacles while all other subject groups were at 5%. If these post-hoc threshold categorizations of subjects as “normal” or “avoiders” are statistically tested via chi-squared, then older women chose to avoid the visible obstacles significantly more often than the other subject groups (p<0.0001) for either threshold value. It should be noted that relatively high values for minimum obstacle distance could be obtained by chance. For example minimum obstacle distance values greater than 30mm were obtained for one older woman and one younger man on the no obstacles surface and for one younger man and one younger woman on the hidden obstacles surface. However, no minimum obstacle distance values greater than 40mm were obtained for any subject on the no obstacles or hidden obstacles surfaces.

### Gait parameter central tendencies

No gait parameter central tendency measure (mean step length, width, or time) significantly differed by subject group. As expected, actual gait speed and step length increased while step time decreased with increasing instructed gait speeds (p<0.0001, Table 1). Step width tended to be greatest when walking slowly and least when walking at preferred speeds, but this effect did not reach significance (p=0.03). Actual gait speed was unaffected by subject group, but slowed on surfaces with obstacles (p=0.0037, p<0.006 for no obstacles vs. visible or hidden obstacles). This effect did not reach the conservative threshold of significance (p<0.0031) when all flooring surfaces were considered, but recoding the data for the presence or absence of obstacles (no obstacles=0 and visible or hidden obstacles=1) revealed that actual gait speed was significantly slower on surfaces with obstacles (p=0.0016).

**Table 1:** Spatiotemporal gait parameter central tendency (mean) and variability (standard deviation) by floor surface and instructed gait speed

**Dependent variable**	**No obstacles floor surface**						**Visible obstacles floor surface**						**Hidden obstacles floor surface**					
	**Slow**		**Preferred**		**Fast**		**Slow**		**Preferred**		**Fast**		**Slow**		**Preferred**		**Fast**	
Central Tendency^a^ [Intersubject Mean (Standard Deviation) of Intrasubject Means]																		
Gait Speed (% Leg Length/s) *	106.9	(30.5)	154.5	(27.6)	249.5	(43.7)	106.1	(26.7)	144.3	(27.5)	228.2	(44.2)	102.9	(27.1)	148.0	(28.1)	223.5	(46.8)
Step Length (% Leg Length) *	71.0	(12.3)	85.6	(10.0)	104.8	(11.4)	71.5	(11.2)	83.5	(10.1)	102.1	(11.6)	68.9	(11.4)	83.8	(11.1)	100.1	(12.4)
Step Width (% Leg Length)	14.6	(3.7)	14.0	(3.1)	13.9	(2.8)	15.2	(3.2)	14.1	(2.9)	14.7	(3.0)	15.2	(3.3)	14.3	(3.2)	14.6	(3.1)
Step Time (s) *	0.69	(0.10)	0.56	(0.05)	0.42	(0.05)	0.70	(0.10)	0.59	(0.06)	0.15	(0.03)	0.70	(0.11)	0.57	(0.05)	0.46	(0.06)
Variability^b^ [Intersubject Mean (Standard Deviation) of Intrasubject Standard Deviations]																		
Gait Speed (% Leg Length/s) *	6.5	(2.6)	7.5	(4.7)	10.6	(7.8)	6.4	(4.0)	6.5	(4.0)	8.9	(6.2)	7.2	(3.7)	6.3	(2.8)	11.2	(6.2)
Step Length (% Leg Length) *	3.9	(1.0)	3.9	(1.3)	4.6	(1.2)	5.5	(3.2)	5.8	(3.8)	5.8	(2.7)	4.5	(1.2)	3.6	(0.8)	4.9	(1.6)
Step Width (% Leg Length) †																		
Younger men	3.8	(1.1)	3.8	(0.8)	4.2	(1.2)	3.6	(1.2)	4.1	(0.8)	4.6	(1.1)	4.2	(1.3)	4.1	(1.0)	4.3	(0.7)
Younger Women	3.4	(0.6)	3.7	(0.8)	4.3	(1.2)	4.3	(1.6)	4.8	(2.0)	4.0	(1.0)	3.7	(1.1)	3.7	(0.7)	4.4	(1.3)
Older Men	3.9	(0.8)	4.1	(1.0)	4.8	(0.8)	4.8	(1.9)	5.3	(1.7)	4.9	(1.5)	3.7	(1.0)	4.5	(0.8)	4.9	(1.3)
Older Women	4.6	(1.8)	5.2	(1.1)	4.7	(1.3)	8.1	(2.7)	8.2	(2.6)	8.9	(3.0)	4.8	(1.4)	5.1	(1.0)	5.3	(0.9)
Step Time (s) *	0.04	(0.03)	0.02	(0.02)	0.02	(0.01)	0.05	(0.03)	0.03	(0.02)	0.02	(0.01)	0.05	(0.03)	0.03	(0.01)	0.02	(0.01)

^a^ Normal distributions tested via repearted-measures linear mixed models.

^b^ Non-normal distributions tested via Kruskall-Wallace.

* p<0.0001 effect of instructed gait speed.

† p<0.0001 effect of subject group.

Data also shown by subject group if effect of subject group significant (p<0.0031).

### Gait parameter variabilities

**Figure 3:**
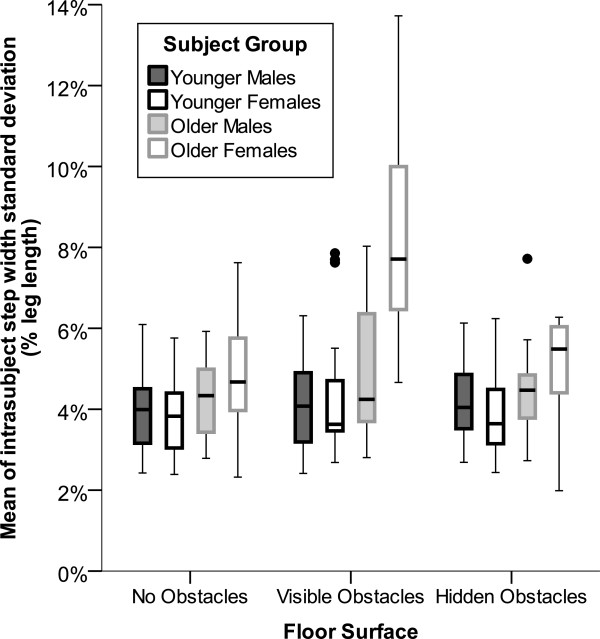
**Error bar plots of step width variability by subject group and flooring condition.** Step width variability was not affected by gait speed but differed significantly by subject group (p<0.0001) and non-significantly by flooring surface (p=0.0066) primarily due to a doubling of step width variability in older females on the visible obstacle surface. While the flooring surface effect did not reach the threshold of significance (p<0.0031) when data were coded for all three surface conditions, when the data were recoded to test for the effects of obstacle visibility (visible obstacles=1 and both other conditions=0), then step width variability was significantly (p<0.0023) increased by obstacle visibility.

**Figure 4:**
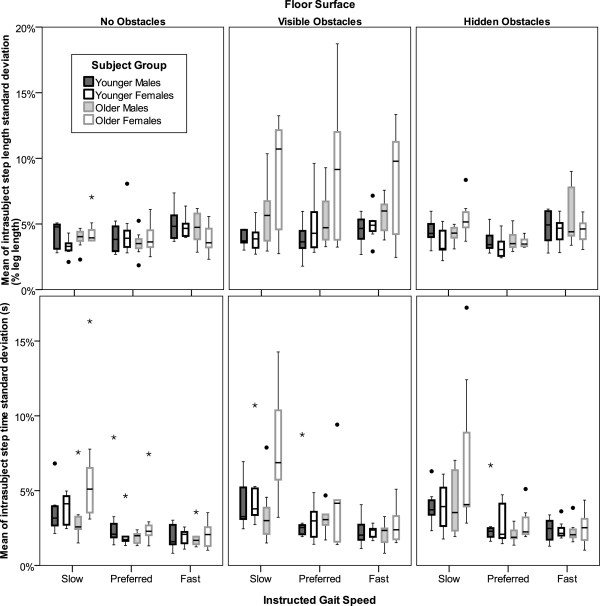
**Error bar plots of step length and time variability by subject group, flooring condition, and speed.** Step length and time variability were greater on surfaces with obstacles, but these effects (p=0.007 and 0.02, respectively) did not reach the threshold of significance (p<0.0031) when data were coded for all three surface conditions. However, when the data were recoded to test for the presence or absence of obstacles (no obstacles=0 and visible or hidden obstacles=1) and for the effects of obstacle visibility (visible obstacles=1 and both other conditions=0), then step length variability was significantly (p<0.0019) increased by obstacle visibility while the presence of obstacles (whether they were visible or not) had a greater effect on step time variability (p=0.009) than obstacle visibility (p=0.05). This effect on step time variability was most extreme for gait at slower instructed speeds (bottom row).

The effects of instructed gait speed on step length and time variability followed those of the mean values, with actual gait speed and step length variability increasing and step time variability decreasing for faster instructed gait speeds. Step width variability was not significantly affected by gait speed (p=0.02) but differed significantly by subject group (p<0.0001) primarily due to a doubling of step width variability in older women on the visible obstacle surface (Figure 3). Step length, width, and time variability were also greater on surfaces with obstacles, but these effects (p=0.007, 0.006, and 0.02; Figures 3 &4) did not reach the threshold of significance (p<0.0031). However, when the data were recoded to test for the effects of obstacle visibility (visible obstacles=1 and both other conditions=0), then variability of step length and width were both significantly (p≤0.0023) increased by obstacle visibility. When the data were recorded to test for the presence or absence of obstacles (no obstacles=0 and visible or hidden obstacles=1) the only effect on gait variability that strengthened from the initial analysis was for step time variability (p=0.009 vs. p=0.02). This effect on step time variability was most pronounced for gait at slower instructed speeds (Figure 4, bottom row).

These effects of flooring surface on gait variability may have been confounded by differences in obstacle avoidance strategy, so an additional analysis was conducted to determine the effects of subject group and gait variability on minimum obstacle distance when walking on the visible obstacles surface. Subject group and variability in step length, width, and time were regressed on minimum obstacle distance. The resulting model explained 65% of the variance in minimum obstacle distance with only step width variability (β=0.54, t=5.0, p<0.0001) and to a lesser extent step length variability (β=0.27, t=2.4, p=0.018) as significant factors in the model. Step width variability alone explained 61% of the variance in minimum obstacle distance, while step length variability alone explained only 36% of this variance and only 3% of additional variance when added to a model including step width variability. If the data from the obstacle “avoiders” as classified by either the 35mm or 45mm threshold are excluded, the strength of these correlations decreases (e.g. R^2^=0.65 for overall model drops to 0.37 for 45mm and 0.14 for 35mm threshold exclusions) but the correlations regarding step width variability remain significant. Note that these correlation results are only for the visible obstacle surface where feedforward control to actively avoid obstacles was possible.

## Discussion

When traversing a floor with multiple small visible obstacles, some healthy older subjects choose to actively avoid obstacles that do not substantially perturb their gait. The older women tested here chose to avoid the obstacles more frequently than the older men, but some older men also adopted this strategy. Thus, this observed gender effect could have been due to other underlying variables that quantify perceived abilities or environmental threat that were either not captured or would have required greater sample sizes to reach significance.

The obstacle avoidance strategy observed here was implemented primarily by modulating step width, but also to a lesser extent by modulating step length as indicated by increases in step width and length variability. However, it is not clear if this obstacle avoidance strategy is appropriate and beneficial or overcautious and maladaptive. This strategy results in fewer obstacles encountered at a consequence of a less efficient gait pattern [18] that has been shown to indicate increased fall risk [9]. While the goal of this obstacle avoidance strategy was likely to increase safety margins and reduce the risk of tripping or stumbling, it may also have been due to a desire to perform the task in a manner the subject believed was desired by the experimenter.

The regression models indicated that foot placement to avoid these small obstacles by healthy adults was proactively regulated primarily by modulating step width and to a lesser extent by modulating step length. Variability in step timing was more related to the presence or absence of obstacles than to obstacle visibility, indicating that temporal gait variability is more reactive than proactive in nature for this task. While increased gait variation could also be due to perturbations induced by stepping on the obstacles, the lack of significant increases in step width and length variability on the hidden obstacles surface as compared to the no obstacles surface indicates that stepping on the small obstacles used did not substantially perturb gait and that the increased variability observed on the visible obstacles surface was due to active obstacle avoidance using feedforward or proactive control of foot placement.

The hypotheses were all partially supported and partially rejected. The first hypothesis (variability of step width and time would be greater on uneven surfaces and step width variability would be greater in the older subjects) was supported with the exception that the increase in step time variability on uneven surfaces did not reach significance. The second hypothesis (subjects would actively avoid visible obstacles) was also partially supported in that some subjects selected this strategy, but most subjects did not. The final hypothesis (gait variability would be greater on the visible obstacles surface) was also partially supported in that step width and length variability were greater on the visible obstacles surface, although step time variability was not.

The effects of increased gait speed on reducing step length and time variability without significantly altering step width variability agrees with earlier findings [10] and could aid in the interpretation of the occasionally contradictory findings of gait variability as a measure of fall risk [4-9]. It should also be noted that increased gait variability is not always a marker of instability, but may be an appropriate adaptation to increasing variability of the environment or task [19]. However, the distinction between “appropriate” and “inappropriate” adaptations to real or perceived environmental challenge is not necessarily clear or easily determined, nor is it necessarily consistent across populations or even individual subjects. This poses a problem for the analysis of data for studies involving gait across visible challenging terrain in a laboratory as well as for studies tracking real-world gait using wearable sensor systems. Such studies should objectively quantify the environment as well as subject performance in order to comprehensively assess the data.

## Conclusions

Some healthy older subjects choose to actively avoid small obstacles that do not substantially perturb their gait by modulating step width and, to a lesser extent, step length. It is not clear if this obstacle avoidance strategy is appropriate and beneficial or overcautious and maladaptive, as it results in fewer obstacles encountered at a consequence of a less efficient gait pattern [18] that has been shown to indicate increased fall risk [9]. Further research is needed on the appropriateness of strategy selection when the environmental demands and/or task requirements have multiple possible completion strategies with conflicting objectives (i.e. perceived safety vs. efficiency).

## Competing interests

The author declares that he has no competing interests.

## Authors´ contributions

BS is solely responsible for this research and manuscript.
